# Crystal structure and Hirshfeld surface analysis of (*Z*)-4-({[2-(benzo[*b*]thio­phen-3-yl)cyclo­pent-1-en-1-yl]meth­yl}(phen­yl)amino)-4-oxobut-2-enoic acid

**DOI:** 10.1107/S2056989024003232

**Published:** 2024-04-26

**Authors:** Elizaveta D. Yakovleva, Evgeniya R. Shelukho, Mikhail S. Grigoriev, Khudayar I. Hasanov, Nurlana D. Sadikhova, Mehmet Akkurt, Ajaya Bhattarai

**Affiliations:** a RUDN University, 6 Miklukho-Maklaya St., Moscow 117198, Russian Federation; b Frumkin Institute of Physical Chemistry and Electrochemistry, Russian Academy of Sciences, Leninskiy prospect 31-4, Moscow 119071, Russian Federation; c Western Caspian University, Istiqlaliyyat Street 31, AZ 1001, Baku, Azerbaijan; d Azerbaijan Medical University, Scientific Research Centre (SRC), A. Kasumzade St. 14, AZ 1022, Baku, Azerbaijan; e Department of Chemistry, Baku State University, Z. Xalilov Str. 23, AZ 1148 Baku, Azerbaijan; f Department of Physics, Faculty of Sciences, Erciyes University, 38039 Kayseri, Türkiye; g Department of Chemistry, M.M.A.M.C. (Tribhuvan University), Biratnagar, Nepal; Institute of Chemistry, Chinese Academy of Sciences

**Keywords:** crystal structure, acyl­ation, thienyl­allyl­amine, maleic acid amide, weak inter­action, Hirshfeld surface analysis

## Abstract

In the title compound, C_24_H_21_NO_3_S, the cyclopentene ring adopts an envelope conformation. In the crystal, mol­ecules are linked by C—H⋯π inter­actions, forming ribbons along the *a* axis. Inter­molecular C—H⋯O hydrogen bonds connect these ribbons to each other, forming layers parallel to the (0  1) plane. The mol­ecular packing is strengthened by van der Waals inter­actions between the layers. The inter­molecular contacts were qu­anti­fied using Hirshfeld surface analysis and two-dimensional fingerprint plots, revealing the relative contributions of the contacts to the crystal packing to be H⋯H 46.0%, C⋯H/H⋯C 21.1%, O⋯H/H⋯O 20.6% and S⋯H/H⋯S 9.0%.

## Abstract 2

In the crystal, mol­ecules are linked by C—H⋯π inter­actions, forming ribbons along the *a* axis. Inter­molecular C—H⋯O hydrogen bonds connect these ribbons to each other, forming layers parallel to the (0 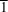 1) plane. The mol­ecular packing is strengthened by van der Waals inter­actions between the layers.

## 1. Chemical context

**Figure 1:**
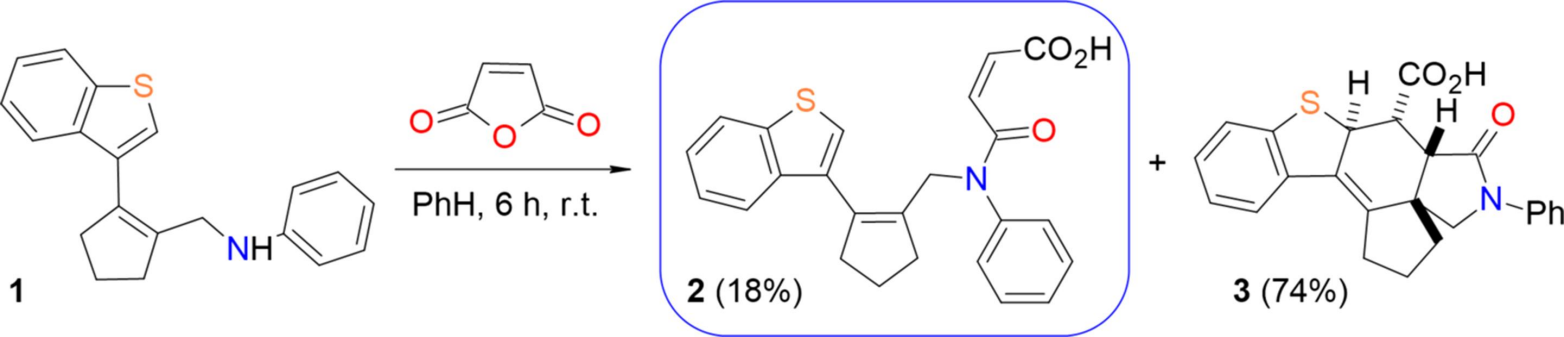
Synthesis of **2**.

Of particular practical value in chemistry are multicomponent approaches based on cyclo­addition reactions, which make it possible to selectively increase the functional periphery around a heterocyclic scaffold in two to four simple steps while achieving high structural and stereochemical diversity of the products. At the same time, of additional inter­est is the strategy of the method for preparing heterocyclic assemblies based on the intra­molecular cyclo­condensation of 3-(hetar­yl)allyl­amines under the action of unsaturated acid anhydrides – the IMDAV reaction (the IntraMolecular Diels–Alder reaction in Vinyl­arenes) (Krishna *et al.*, 2022 ▸). This work is a continuation of studies on the mechanism of the tandem acyl­ation/[4 + 2]-cyclo­addition reaction between 3-(hetar­yl)allyl­amines and maleic anhydride as an example of the IMDAV approach (Horak *et al.*, 2015 ▸, 2017 ▸; Nadirova *et al.*, 2020 ▸; Zubkov *et al.*, 2016 ▸; Yakovleva *et al.*, 2024 ▸). On the other hand, functionalization of amines with multiple coordination centres can be used as an important synthetic strategy for the preparation of new functional materials (Akbari Afkhami *et al.*, 2017 ▸; Abdelhamid *et al.*, 2011 ▸; Khalilov *et al.*, 2021 ▸; Safavora *et al.*, 2019 ▸). In fact, those substituents or functional groups can participate in various sorts of inter­molecular inter­actions (Gurbanov *et al.*, 2018 ▸, 2020 ▸, 2022*a* ▸,*b* ▸; Kopylovich *et al.*, 2011*a* ▸,*b* ▸,*c* ▸; Mahmudov *et al.*, 2013 ▸, 2021 ▸), which improve the function of supra­molecular networks. The co-operation of weak inter­actions with the coordination bond in N-donating ligands can be used in the crystal engineering of tectons (Aliyeva *et al.*, 2024 ▸; Mahmoudi *et al.*, 2017*a* ▸,*b* ▸, 2019 ▸, 2021 ▸). Benzothienyl­allyl­amine **1** (Yakovleva *et al.*, 2024 ▸) is able to readily react with maleic anhydride providing a mixture of products **2** and **3** in nearly qu­anti­tative yield. The synthesis and spectral data for the major adduct **3** have been published previously (Yakovleva *et al.*, 2024 ▸), but the minor amide **2** could not be isolated and characterized because of its high tendency to spontaneously cyclize with the formation of **3** (Fig. 1 ▸). In this work, under mild reaction conditions, we successfully isolated and characterized the inter­mediate maleic amide **2**. Detection of amide **2** confirms directly an assumption that the IMDAV reaction begins with an acyl­ation step followed by an intra­molecular [4 + 2]-cyclo­addition. 
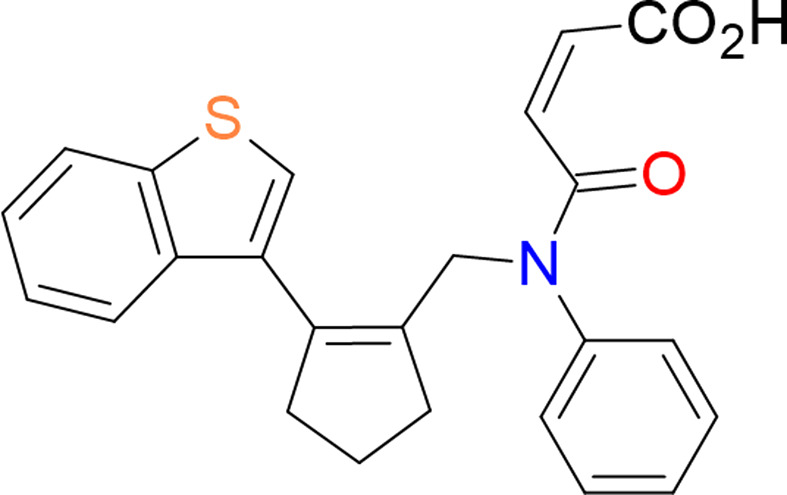


## 2. Structural commentary

**Figure 2:**
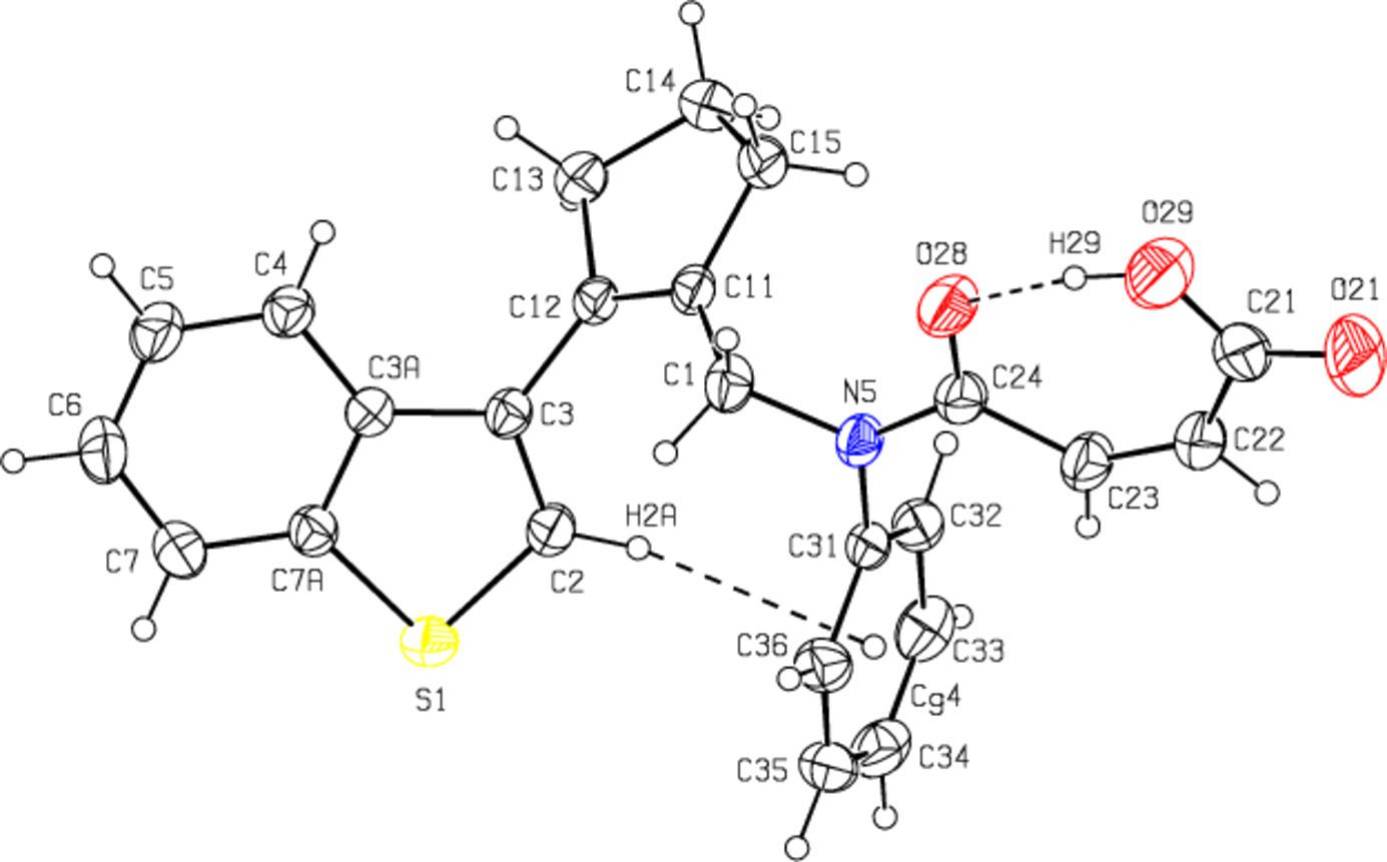
The mol­ecular structure of the title compound, showing the atom labelling and displacement ellipsoids at the 30% probability level.

As can be seen in Fig. 2 ▸, the nine-membered ring system (S1/C2/C3/C3*A*/C4-C7/C7*A*) of the mol­ecule is essentially planar (r.m.s. deviation = 0.002 Å), while the cyclopentene ring (C11–C15) adopts an envelope conformation, with the C14 atom as the flap [puckering parameters (Cremer & Pople, 1975 ▸) are *Q*(2) = 0.200 (3) Å and φ(2) = 103.3 (7)°]. The nine-membered ring system makes an angle of 66.00 (11)° with the r.m.s. plane of the cyclopentene ring. These planes make angles of 61.68 (10) and 64.83 (12)° with the phenyl ring, respectively. The C12—C11—C1—N5, C11—C1—N5—C24, C11—C1—N5—C31, N5—C24—C23—C22, O28—C24—C23—C22, C23—C22—C21—O2 and C23—C22—C21—O29 torsion angles are 117.2 (2), 102.2 (2), −80.0 (2), −177.6 (2), 3.1 (4), −179.9 (3) and 0.3 (5)°, respectively. The bond length and angle values of the title mol­ecule are comparable to those of the mol­ecules in the *Database survey* section.

## 3. Supra­molecular features and Hirshfeld surface analysis

**Figure 3:**
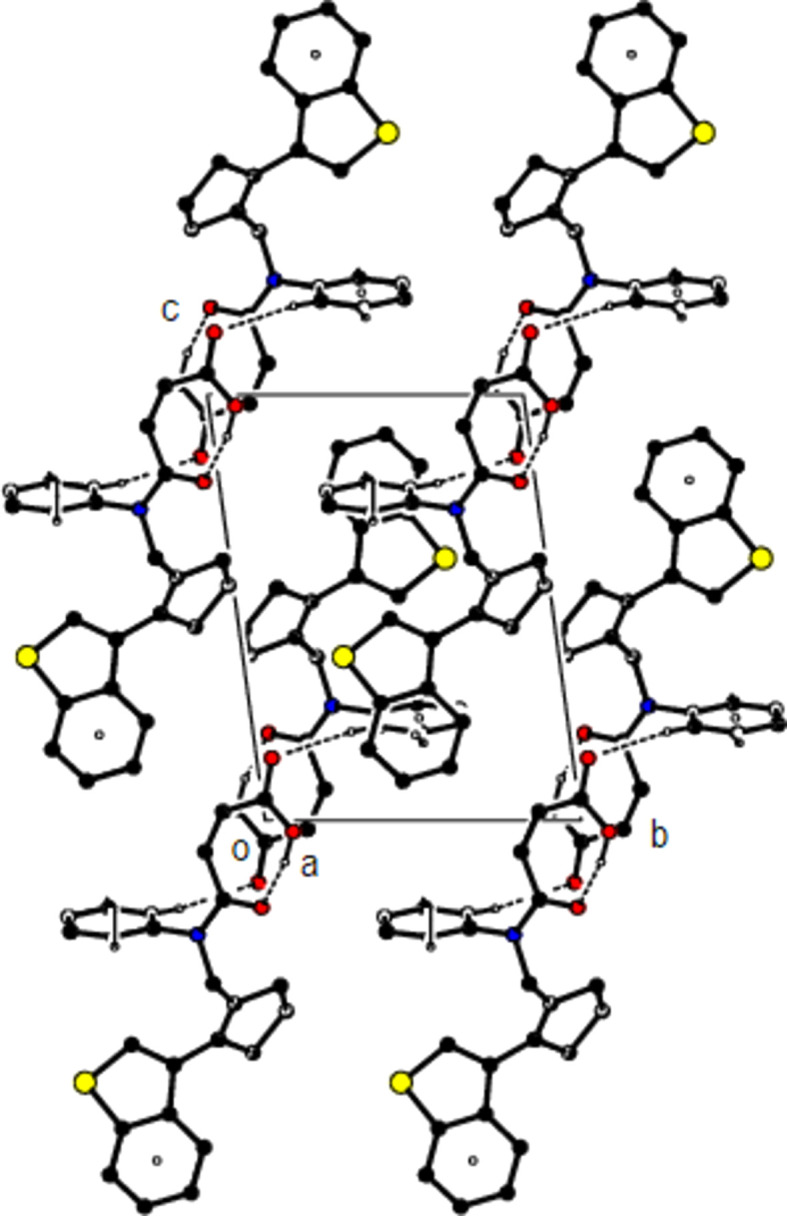
The crystal packing along the *a* axis, showing O—H⋯O, C—H⋯O and C—H⋯π inter­actions.

The mol­ecular conformation remains stable *via* an intra­molecular O29—H29⋯O28 hydrogen bond, which forms a ring with an *S*(7) motif, and an intra­molecular C2—H2*A*⋯*Cg*4 inter­action (Table 1 ▸ and Fig. 2 ▸) (Bernstein *et al.*, 1995 ▸; *Cg*4 is the centroid of the C31–C36 ring). In the crystal, mol­ecules are linked by C—H⋯π inter­actions, forming ribbons along the *a* axis. Inter­molecular C—H⋯O hydrogen bonds connect these ribbons to each other, forming layers parallel to the (0 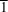 1) plane. The mol­ecular packing is strengthened by van der Waals inter­actions between the layers (Table 1 ▸ and Figs. 3 ▸, 4 ▸ and 5 ▸).

**Figure 6:**
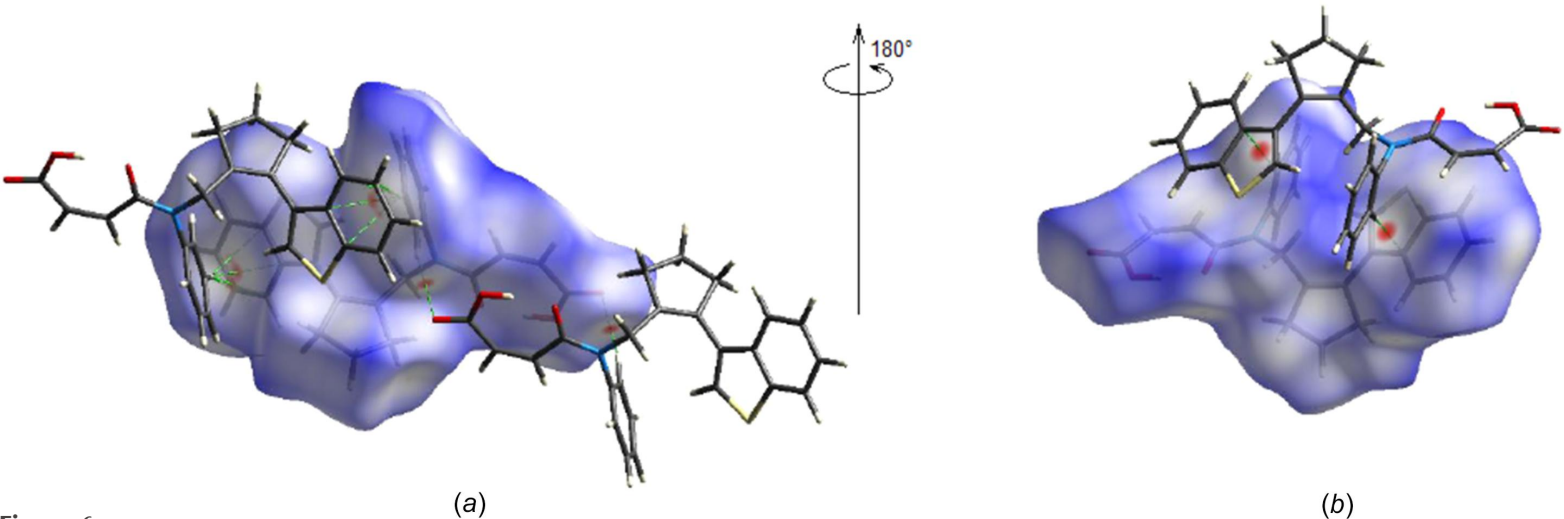
Front (*a*) and back (*b*) views of the three-dimensional Hirshfeld surface, with some C—H⋯O, O—H⋯O and C—H⋯π inter­actions shown.

*CrystalExplorer17.5* (Spackman *et al.*, 2021 ▸) was used to compute the Hirshfeld surfaces and the two-dimensional fingerprints of the title mol­ecule. The *d*_norm_ mappings were performed in the range from −0.1088 (red) to +1.5482 (blue) a.u., on the *d*_norm_ surfaces, allowing the location of the C—H⋯O and C—H⋯π inter­actions (Table 1 ▸ and Fig. 6 ▸).

**Figure 7:**
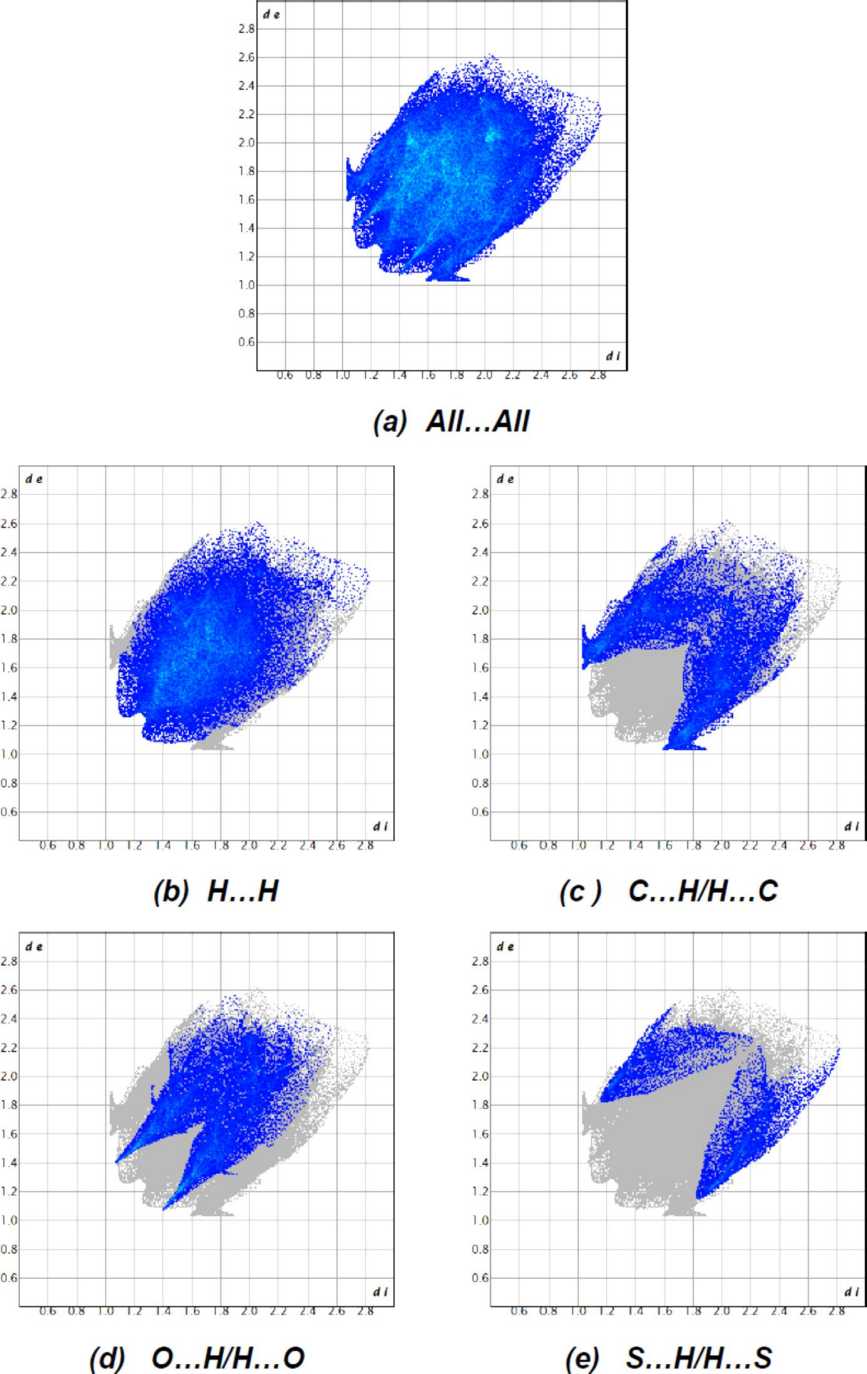
The two-dimensional fingerprint plots for the title mol­ecules showing (*a*) all inter­actions, and delineated into (*b*) H⋯H, (*c*) C⋯H/H⋯C, (*d*) O⋯H/H⋯O and (*e*) S⋯H/H⋯S inter­actions. The *d*_i_ and *d*_e_ values are the closest inter­nal and external distances (in Å) from given points on the Hirshfeld surface.

The fingerprint plots (Fig. 7 ▸) show that H⋯H [Fig. 7 ▸(*b*); 46.0%], C⋯H/H⋯C [Fig. 7 ▸(*c*); 21.1%], O⋯H/H⋯O [Fig. 7 ▸(*d*); 20.6%] and S⋯H/H⋯S [Fig. 7 ▸(*e*); 9.0%] inter­actions have the greatest contributions to the surface contacts. The crystal packing is additionally influenced by C⋯C (2.2%), O⋯O (0.4%), O⋯C/C⋯O (0.3%), N⋯C/C⋯C (0.2%), S⋯C/C⋯S (0.1%) and S⋯O/O⋯S (0.1%) inter­actions. The large number of H⋯H, C⋯H/H⋯C, O⋯H/H⋯O and S⋯H/H⋯S inter­actions indicates that van der Waals inter­actions and hydrogen bonding are important in the crystal packing (Hathwar *et al.*, 2015 ▸).

## 4. Database survey

A search of the Cambridge Structural Database (CSD, Version 5.43, last update November 2022; Groom *et al.*, 2016 ▸) for the 1-benzo­thio­phene unit yielded three compounds related to the title compound, *viz*. CSD refcode WOJBII (Kaur *et al.*, 2014 ▸), GAPZOO (Inaç *et al.*, 2012 ▸) and EYISEK (Sonar *et al.*, 2004 ▸).

In WOJBII, an intra­molecular N—H⋯O hydrogen bond generates an *S*(6) ring. In the crystal, very weak aromatic π–π stacking inter­actions [centroid–centroid separation = 3.9009 (10) Å] are observed. In GAPZOO, the mol­ecular conformation features a short C—H⋯N contact. There are no significant inter­molecular contacts. In EYISEK, inter­molecular hydrogen bonding exists between the imino H atom and the Cl atoms, and gives rise to chains of mol­ecules extending in the *c* direction. Van der Waals forces contribute to the stabilization of the crystal structure.

## 5. Synthesis and crystallization

Maleic anhydride (0.12 g, 1.3 mmol) was added to a solution of the corresponding allyl­amine **1** (0.37 g, 1.2 mmol) in benzene (10 ml). The resulting mixture was stirred for 6 h at room temperature. The resulting precipitate was filtered off, washed with benzene (5 ml), diethyl ether (2 × 5 ml) and air dried to give acid **3** (0.27 g, 74%) as a colourless solid (for full characteristics, see Yakovleva *et al.*, 2024 ▸). The mother liquor was mixed with C_2_H_5_OH (5 ml) and the precipitate was filtered off, washed with benzene (5 ml), diethyl ether (2 × 5 ml) and air dried to give the title compound **2** as a colourless powder (yield 18%, 0.09 g; m.p. 400–402 K). IR (KBr), ν (cm^−1^): 3032 (OH), 1734 (CO_2_), 1669 (N—C=O). ^1^H NMR (600.2 MHz, DMSO-*d*_6_, 298 K): δ (*J*, Hz) 12.71 (*s*, 1H, CO_2_H), 7.93 (*d*, *J* = 8.1, 1H, H-Ar), 7.35–7.09 (*m*, 8H, H-Ar), 6.84 (*s*, 1H, H-2 benzo­thio­phene), 6.37 (*d*, *J* = 12.1, 1H, H-2 CH=CH), 5.71 (*d*, *J* = 12.1, 1H, H-2 CH=CH), 4.36 (*br s*, 2H, NCH_2_), 2.66–2.62 (*m*, 4H, H-3, H-5), 1.93 (*pent*, *J* = 7.6, 2H, H-4). ^13^C {^1^H} NMR (150.9 MHz, DMSO-*d*_6_, 298 K): δ 166.0, 162.0, 140.4, 139.1, 138.5, 136.2, 135.2, 133.3, 132.1, 128.9 (2C), 128.0, 124.4, 124.3, 123.2, 122.8, 122.2, 119.0, 118.0 (2C), 52.9, 34.0, 31.7, 21.1. MS (ESI) *m*/*z*: [*M* + H]^+^ 404. Elemental analysis calculated (%) for C_24_H_21_NO_3_S: C 71.44, H 5.25, N 3.47, S 7.95; found: C 71.40, H 5.35, N 3.55, S, 7.91.

## 6. Refinement

Crystal data, data collection and structure refinement details are summarized in Table 2 ▸. All C-bound H atoms were positioned geometrically (C—H = 0.93 and 0.97 Å) and refined using a riding model with *U*_iso_(H) = 1.2*U*_eq_(C). The O-bound H atom was located in difference Fourier maps [O29—H29 = 0.93 (4) Å] and refined freely.

## Supplementary Material

Crystal structure: contains datablock(s) I, global. DOI: 10.1107/S2056989024003232/nx2009sup1.cif

Structure factors: contains datablock(s) I. DOI: 10.1107/S2056989024003232/nx2009Isup2.hkl

Supporting information file. DOI: 10.1107/S2056989024003232/nx2009Isup3.cml

CCDC reference: 2348265

Additional supporting information:  crystallographic information; 3D view; checkCIF report

## Figures and Tables

**Figure 4:**
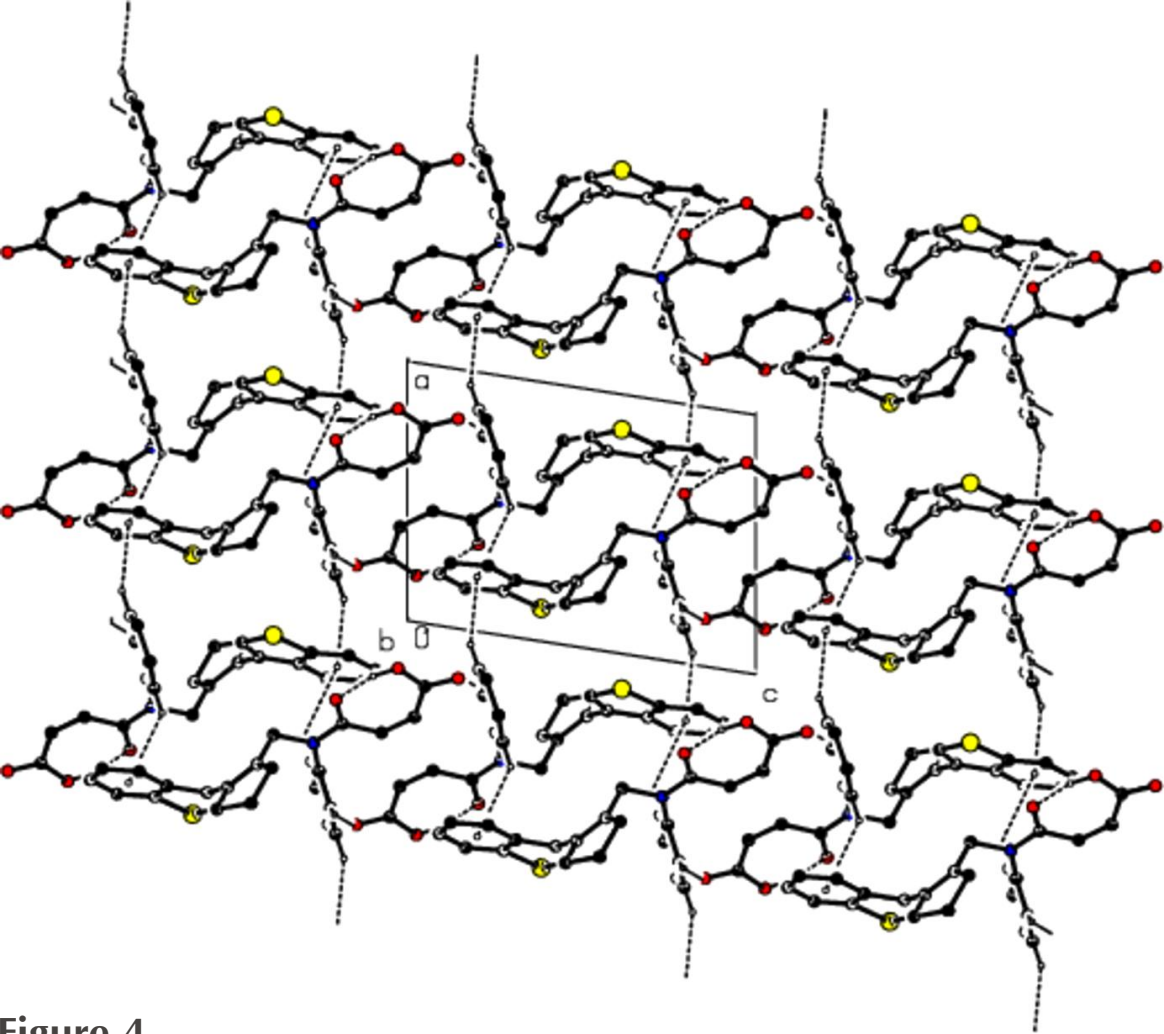
The crystal packing along the *b* axis, showing the O—H⋯O, C—H⋯O and C—H⋯π inter­actions.

**Figure 5:**
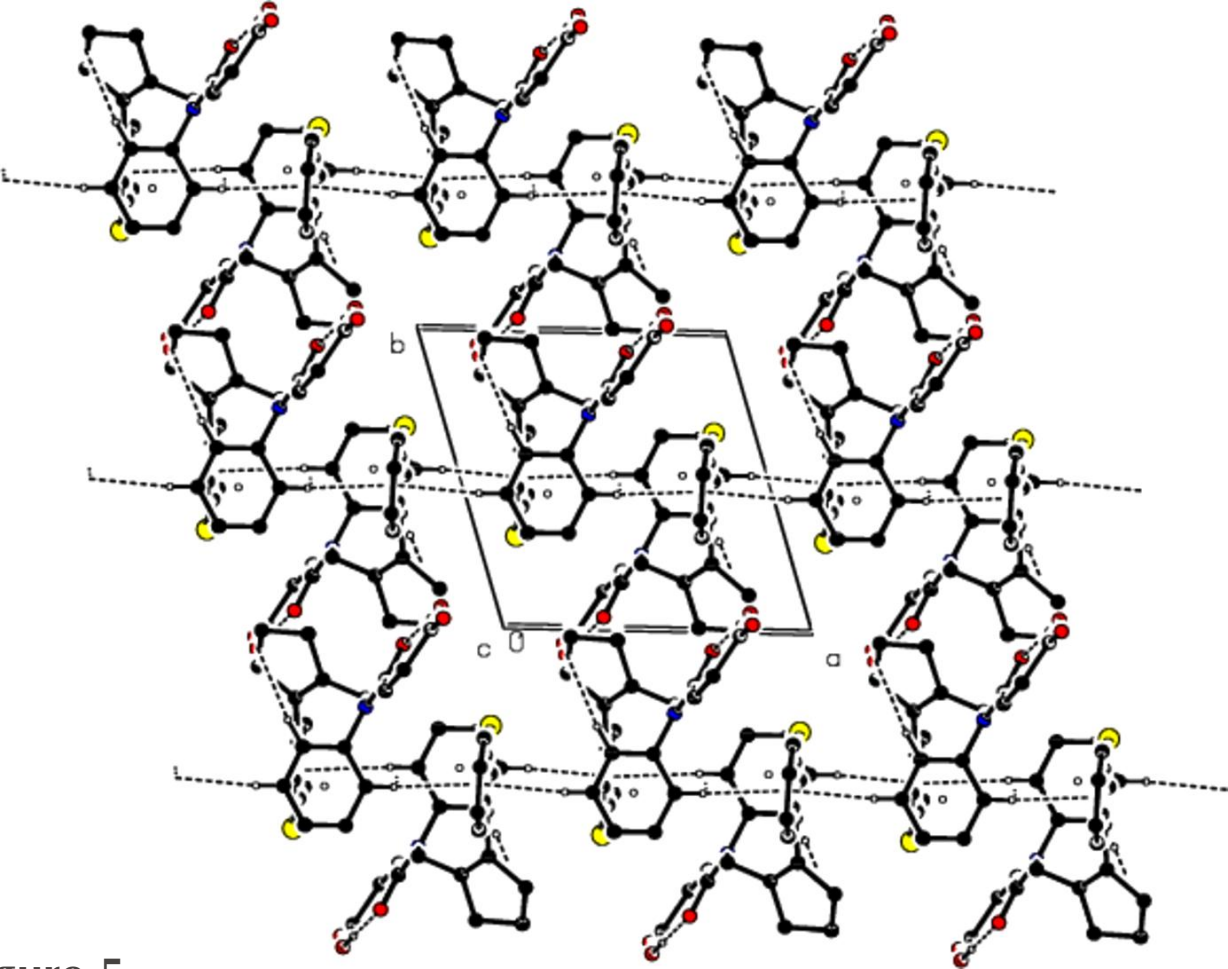
The crystal packing along the *c* axis, showing the O—H⋯O, C—H⋯O and C—H⋯π inter­actions.

**Table 1:** Hydrogen-bond geometry (Å, °) *Cg*3 and *Cg*4 are the centroids of the benzene ring (C3*A*/C4–C7/C7*A*) of the nine-membered ring system (S1/C2-C3/C3*A*/C4–C7/C7*A*) and the phenyl ring (C31–C36), respectively.

*D*—H⋯*A*	*D*—H	H⋯*A*	*D*⋯*A*	*D*—H⋯*A*
O29—H29⋯O28	0.93 (4)	1.60 (4)	2.510 (3)	164 (4)
C32—H32*A*⋯O21^i^	0.93	2.63	3.556 (3)	171
C2—H2*A*⋯*Cg*4	0.93	2.72	3.579 (2)	154
C33—H33*A*⋯*Cg*3^ii^	0.93	2.54	3.408 (3)	155
C36—H36*A*⋯*Cg*3^iii^	0.93	2.79	3.535 (3)	138

Symmetry codes: (i) 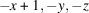 ; (ii) 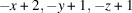 ; (iii) 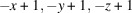 .

**Table 2:** Experimental details

Crystal data	
Chemical formula	C_24_H_21_NO_3_S
*M*_r_	403.48
Crystal system, space group	Triclinic, *P* 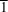
Temperature (K)	296
*a*, *b*, *c* (Å)	9.4270 (5), 9.4386 (4), 12.3849 (7)
α, β, γ (°)	95.392 (3), 96.849 (3), 106.512 (3)
*V* (Å^3^)	1039.49 (9)
*Z*	2
Radiation type	Mo *K*α
μ (mm^−1^)	0.18
Crystal size (mm)	0.32 × 0.26 × 0.22

Data collection	
Diffractometer	Bruker Kappa APEXII area-detector
Absorption correction	Multi-scan (*SADABS*; Bruker, 2008 ▸)
*T*_min_, *T*_max_	0.882, 0.961
No. of measured, independent and observed [*I* > 2σ(*I*)] reflections	13624, 4758, 2579
*R*_int_	0.046
(sin θ/λ)_max_ (Å^−1^)	0.650

Refinement	
*R*[*F* ^2^ > 2σ(*F* ^2^)], *wR*(*F* ^2^), *S*	0.049, 0.115, 0.99
No. of reflections	4758
No. of parameters	266
H-atom treatment	H atoms treated by a mixture of independent and constrained refinement
Δρ_max_, Δρ_min_ (e Å^−3^)	0.21, −0.24

Computer programs: *APEX4* and *SAINT* (Bruker, 2018 ▸), *SHELXT* (Sheldrick, 2015*a* ▸), *SHELXL2018* (Sheldrick, 2015*b* ▸), *ORTEP-3 for Windows* (Farrugia, 2012 ▸) and *PLATON* (Spek, 2020 ▸).

## supplementary crystallographic information

### Crystal data

**Table:**

C_24_H_21_NO_3_S	*Z* = 2
*M**_r_* = 403.48	*F*(000) = 424
Triclinic, *P*1	*D*_x_ = 1.289 Mg m^−^^3^
*a* = 9.4270 (5) Å	Mo *K*α radiation, λ = 0.71073 Å
*b* = 9.4386 (4) Å	Cell parameters from 2139 reflections
*c* = 12.3849 (7) Å	θ = 2.7–21.1°
α = 95.392 (3)°	µ = 0.18 mm^−^^1^
β = 96.849 (3)°	*T* = 296 K
γ = 106.512 (3)°	Fragment, colourless
*V* = 1039.49 (9) Å^3^	0.32 × 0.26 × 0.22 mm

### Data collection

**Table:**

Bruker Kappa APEXII area-detector diffractometer	2579 reflections with *I* > 2σ(*I*)
φ and ω scans	*R*_int_ = 0.046
Absorption correction: multi-scan (SADABS; Bruker, 2008)	θ_max_ = 27.5°, θ_min_ = 4.3°
*T*_min_ = 0.882, *T*_max_ = 0.961	*h* = −12→12
13624 measured reflections	*k* = −12→12
4758 independent reflections	*l* = −16→16

### Refinement

**Table:**

Refinement on *F*^2^	0 restraints
Least-squares matrix: full	Hydrogen site location: mixed
*R*[*F*^2^ > 2σ(*F*^2^)] = 0.049	H atoms treated by a mixture of independent and constrained refinement
*wR*(*F*^2^) = 0.115	*w* = 1/[σ^2^(*F*_o_^2^) + (0.0397*P*)^2^ + 0.1315*P*] where *P* = (*F*_o_^2^ + 2*F*_c_^2^)/3
*S* = 0.99	(Δ/σ)_max_ < 0.001
4758 reflections	Δρ_max_ = 0.21 e Å^−^^3^
266 parameters	Δρ_min_ = −0.24 e Å^−^^3^

### Special details

**Table:**

Geometry. All esds (except the esd in the dihedral angle between two l.s. planes) are estimated using the full covariance matrix. The cell esds are taken into account individually in the estimation of esds in distances, angles and torsion angles; correlations between esds in cell parameters are only used when they are defined by crystal symmetry. An approximate (isotropic) treatment of cell esds is used for estimating esds involving l.s. planes.

### Fractional atomic coordinates and isotropic or equivalent isotropic displacement parameters (Å^2^)

**Table:**

	*x*	*y*	*z*	*U*_iso_*/*U*_eq_	
S1	0.87037 (7)	0.68170 (6)	0.61573 (5)	0.0594 (2)	
O21	0.1824 (3)	−0.0483 (2)	−0.14619 (18)	0.1267 (9)	
O28	0.3378 (2)	0.04844 (18)	0.20444 (14)	0.0791 (6)	
O29	0.1793 (3)	−0.0840 (2)	0.0262 (2)	0.1150 (9)	
H29	0.224 (5)	−0.045 (4)	0.098 (3)	0.158 (16)*	
N5	0.51977 (19)	0.26204 (18)	0.26864 (14)	0.0485 (5)	
C1	0.5116 (2)	0.2342 (2)	0.38357 (17)	0.0521 (6)	
H1A	0.415584	0.163072	0.386915	0.063*	
H1B	0.517549	0.326562	0.428083	0.063*	
C2	0.8233 (2)	0.5180 (2)	0.52668 (18)	0.0512 (6)	
H2A	0.830032	0.515746	0.452274	0.061*	
C3A	0.7750 (2)	0.4286 (2)	0.68961 (17)	0.0406 (5)	
C3	0.7756 (2)	0.3932 (2)	0.57416 (17)	0.0424 (5)	
C4	0.7341 (2)	0.3332 (2)	0.76803 (19)	0.0500 (6)	
H4A	0.699452	0.230472	0.747835	0.060*	
C5	0.7460 (3)	0.3934 (3)	0.8750 (2)	0.0671 (7)	
H5A	0.718992	0.330231	0.927272	0.080*	
C6	0.7974 (3)	0.5468 (3)	0.9073 (2)	0.0763 (8)	
H6A	0.805046	0.584341	0.980612	0.092*	
C7A	0.8246 (2)	0.5832 (2)	0.72381 (18)	0.0466 (5)	
C7	0.8367 (3)	0.6423 (3)	0.8327 (2)	0.0630 (7)	
H7A	0.870953	0.744824	0.854184	0.076*	
C11	0.6337 (2)	0.1755 (2)	0.43046 (17)	0.0436 (5)	
C12	0.7407 (2)	0.2407 (2)	0.51466 (16)	0.0426 (5)	
C13	0.8318 (3)	0.1381 (2)	0.54573 (19)	0.0552 (6)	
H13A	0.806912	0.098031	0.612696	0.066*	
H13B	0.938174	0.189892	0.555652	0.066*	
C14	0.7866 (3)	0.0148 (2)	0.4478 (2)	0.0598 (6)	
H14A	0.861755	0.031363	0.399435	0.072*	
H14B	0.775219	−0.081812	0.472422	0.072*	
C15	0.6382 (3)	0.0221 (2)	0.38884 (19)	0.0554 (6)	
H15A	0.635418	0.009946	0.309899	0.066*	
H15B	0.554836	−0.054219	0.407358	0.066*	
C21	0.2336 (4)	−0.0081 (3)	−0.0502 (3)	0.0835 (9)	
C22	0.3591 (3)	0.1295 (3)	−0.0208 (2)	0.0683 (7)	
H22A	0.388556	0.174848	−0.081361	0.082*	
C23	0.4395 (3)	0.2030 (3)	0.07372 (19)	0.0645 (7)	
H23A	0.513533	0.290349	0.068407	0.077*	
C24	0.4279 (3)	0.1656 (2)	0.18604 (19)	0.0556 (6)	
C31	0.6267 (2)	0.3994 (2)	0.25315 (16)	0.0423 (5)	
C32	0.7609 (3)	0.3991 (2)	0.22026 (18)	0.0520 (6)	
H32A	0.784507	0.310332	0.207993	0.062*	
C33	0.8601 (3)	0.5327 (3)	0.2057 (2)	0.0636 (7)	
H33A	0.949957	0.533354	0.181491	0.076*	
C34	0.8276 (3)	0.6634 (3)	0.2264 (2)	0.0708 (8)	
H34A	0.895368	0.752805	0.216795	0.085*	
C35	0.6957 (4)	0.6629 (3)	0.2613 (2)	0.0696 (8)	
H35A	0.674317	0.752324	0.276532	0.083*	
C36	0.5938 (3)	0.5303 (2)	0.2740 (2)	0.0593 (6)	
H36A	0.503131	0.530002	0.296694	0.071*	

### Atomic displacement parameters (Å^2^)

**Table:**

	*U*^11^	*U*^22^	*U*^33^	*U*^12^	*U*^13^	*U*^23^
S1	0.0715 (4)	0.0433 (3)	0.0593 (4)	0.0106 (3)	0.0078 (3)	0.0104 (3)
O21	0.177 (2)	0.0925 (15)	0.0651 (15)	−0.0041 (15)	−0.0275 (15)	−0.0161 (12)
O28	0.0936 (13)	0.0594 (10)	0.0586 (12)	−0.0113 (10)	−0.0108 (10)	0.0158 (9)
O29	0.155 (2)	0.0688 (13)	0.0749 (16)	−0.0215 (13)	−0.0289 (15)	0.0109 (12)
N5	0.0521 (11)	0.0506 (10)	0.0382 (11)	0.0113 (9)	−0.0011 (9)	0.0063 (8)
C1	0.0502 (14)	0.0593 (13)	0.0408 (14)	0.0087 (11)	0.0033 (11)	0.0057 (11)
C2	0.0588 (14)	0.0500 (12)	0.0416 (13)	0.0111 (11)	0.0064 (11)	0.0088 (10)
C3A	0.0347 (11)	0.0445 (11)	0.0423 (13)	0.0121 (9)	0.0037 (9)	0.0067 (9)
C3	0.0408 (12)	0.0462 (11)	0.0382 (13)	0.0111 (10)	0.0022 (10)	0.0069 (9)
C4	0.0525 (14)	0.0518 (12)	0.0486 (15)	0.0157 (11)	0.0138 (11)	0.0124 (11)
C5	0.0861 (19)	0.0754 (17)	0.0500 (17)	0.0312 (15)	0.0246 (14)	0.0196 (13)
C6	0.106 (2)	0.0870 (19)	0.0440 (16)	0.0427 (18)	0.0152 (15)	−0.0004 (14)
C7A	0.0470 (13)	0.0486 (12)	0.0440 (14)	0.0162 (10)	0.0031 (10)	0.0043 (10)
C7	0.0771 (18)	0.0578 (14)	0.0537 (17)	0.0250 (13)	0.0048 (14)	−0.0032 (13)
C11	0.0491 (13)	0.0439 (11)	0.0344 (12)	0.0075 (10)	0.0067 (10)	0.0075 (9)
C12	0.0490 (13)	0.0414 (10)	0.0356 (12)	0.0090 (10)	0.0088 (10)	0.0082 (9)
C13	0.0633 (15)	0.0535 (12)	0.0488 (15)	0.0192 (12)	0.0022 (12)	0.0097 (11)
C14	0.0716 (17)	0.0466 (12)	0.0604 (16)	0.0185 (12)	0.0070 (13)	0.0048 (11)
C15	0.0676 (16)	0.0461 (12)	0.0449 (14)	0.0082 (11)	0.0034 (12)	0.0025 (10)
C21	0.119 (3)	0.0541 (15)	0.060 (2)	0.0158 (16)	−0.0188 (19)	−0.0045 (15)
C22	0.091 (2)	0.0615 (15)	0.0466 (16)	0.0204 (14)	−0.0031 (14)	0.0054 (12)
C23	0.0765 (18)	0.0586 (14)	0.0455 (16)	0.0042 (13)	−0.0044 (13)	0.0097 (12)
C24	0.0632 (15)	0.0500 (13)	0.0474 (15)	0.0120 (12)	−0.0043 (12)	0.0070 (11)
C31	0.0476 (13)	0.0441 (11)	0.0350 (12)	0.0171 (10)	−0.0016 (10)	0.0041 (9)
C32	0.0549 (15)	0.0567 (13)	0.0479 (14)	0.0254 (12)	0.0006 (12)	0.0062 (11)
C33	0.0462 (14)	0.0824 (18)	0.0582 (17)	0.0130 (13)	0.0005 (12)	0.0180 (14)
C34	0.075 (2)	0.0585 (16)	0.0597 (18)	−0.0027 (14)	−0.0144 (15)	0.0168 (13)
C35	0.095 (2)	0.0478 (14)	0.0624 (18)	0.0272 (15)	−0.0095 (16)	0.0012 (12)
C36	0.0627 (15)	0.0587 (14)	0.0608 (16)	0.0286 (13)	0.0033 (13)	0.0051 (12)

### Geometric parameters (Å, º)

**Table:**

S1—C2	1.725 (2)	C11—C15	1.505 (3)
S1—C7A	1.728 (2)	C12—C13	1.512 (3)
O21—C21	1.210 (3)	C13—C14	1.529 (3)
O28—C24	1.247 (3)	C13—H13A	0.9700
O29—C21	1.305 (4)	C13—H13B	0.9700
O29—H29	0.93 (4)	C14—C15	1.522 (3)
N5—C24	1.338 (3)	C14—H14A	0.9700
N5—C31	1.443 (3)	C14—H14B	0.9700
N5—C1	1.478 (3)	C15—H15A	0.9700
C1—C11	1.495 (3)	C15—H15B	0.9700
C1—H1A	0.9700	C21—C22	1.468 (4)
C1—H1B	0.9700	C22—C23	1.329 (3)
C2—C3	1.352 (3)	C22—H22A	0.9300
C2—H2A	0.9300	C23—C24	1.476 (3)
C3A—C4	1.399 (3)	C23—H23A	0.9300
C3A—C7A	1.406 (3)	C31—C36	1.367 (3)
C3A—C3	1.439 (3)	C31—C32	1.375 (3)
C3—C12	1.482 (3)	C32—C33	1.382 (3)
C4—C5	1.370 (3)	C32—H32A	0.9300
C4—H4A	0.9300	C33—C34	1.362 (3)
C5—C6	1.391 (3)	C33—H33A	0.9300
C5—H5A	0.9300	C34—C35	1.363 (4)
C6—C7	1.361 (4)	C34—H34A	0.9300
C6—H6A	0.9300	C35—C36	1.380 (4)
C7A—C7	1.389 (3)	C35—H35A	0.9300
C7—H7A	0.9300	C36—H36A	0.9300
C11—C12	1.326 (3)		
			
C2—S1—C7A	90.81 (10)	C14—C13—H13B	111.1
C21—O29—H29	116 (2)	H13A—C13—H13B	109.0
C24—N5—C31	123.48 (18)	C15—C14—C13	105.85 (18)
C24—N5—C1	120.39 (19)	C15—C14—H14A	110.6
C31—N5—C1	116.08 (16)	C13—C14—H14A	110.6
N5—C1—C11	112.78 (18)	C15—C14—H14B	110.6
N5—C1—H1A	109.0	C13—C14—H14B	110.6
C11—C1—H1A	109.0	H14A—C14—H14B	108.7
N5—C1—H1B	109.0	C11—C15—C14	103.55 (17)
C11—C1—H1B	109.0	C11—C15—H15A	111.1
H1A—C1—H1B	107.8	C14—C15—H15A	111.1
C3—C2—S1	114.34 (17)	C11—C15—H15B	111.1
C3—C2—H2A	122.8	C14—C15—H15B	111.1
S1—C2—H2A	122.8	H15A—C15—H15B	109.0
C4—C3A—C7A	118.37 (19)	O21—C21—O29	121.5 (3)
C4—C3A—C3	129.57 (19)	O21—C21—C22	118.3 (3)
C7A—C3A—C3	112.06 (18)	O29—C21—C22	120.2 (2)
C2—C3—C3A	111.32 (18)	C23—C22—C21	133.7 (3)
C2—C3—C12	123.2 (2)	C23—C22—H22A	113.2
C3A—C3—C12	125.29 (18)	C21—C22—H22A	113.2
C5—C4—C3A	119.1 (2)	C22—C23—C24	129.1 (2)
C5—C4—H4A	120.5	C22—C23—H23A	115.5
C3A—C4—H4A	120.5	C24—C23—H23A	115.5
C4—C5—C6	121.6 (2)	O28—C24—N5	120.6 (2)
C4—C5—H5A	119.2	O28—C24—C23	122.1 (2)
C6—C5—H5A	119.2	N5—C24—C23	117.3 (2)
C7—C6—C5	120.7 (2)	C36—C31—C32	120.5 (2)
C7—C6—H6A	119.7	C36—C31—N5	118.8 (2)
C5—C6—H6A	119.7	C32—C31—N5	120.68 (18)
C7—C7A—C3A	121.8 (2)	C31—C32—C33	119.0 (2)
C7—C7A—S1	126.71 (17)	C31—C32—H32A	120.5
C3A—C7A—S1	111.46 (16)	C33—C32—H32A	120.5
C6—C7—C7A	118.5 (2)	C34—C33—C32	120.6 (2)
C6—C7—H7A	120.8	C34—C33—H33A	119.7
C7A—C7—H7A	120.8	C32—C33—H33A	119.7
C12—C11—C1	126.95 (18)	C33—C34—C35	119.9 (2)
C12—C11—C15	112.06 (18)	C33—C34—H34A	120.1
C1—C11—C15	120.81 (18)	C35—C34—H34A	120.1
C11—C12—C3	127.71 (18)	C34—C35—C36	120.3 (2)
C11—C12—C13	111.02 (17)	C34—C35—H35A	119.8
C3—C12—C13	121.27 (17)	C36—C35—H35A	119.8
C12—C13—C14	103.43 (17)	C31—C36—C35	119.6 (2)
C12—C13—H13A	111.1	C31—C36—H36A	120.2
C14—C13—H13A	111.1	C35—C36—H36A	120.2
C12—C13—H13B	111.1		
			
C24—N5—C1—C11	102.2 (2)	C2—C3—C12—C13	115.1 (2)
C31—N5—C1—C11	−80.0 (2)	C3A—C3—C12—C13	−59.9 (3)
C7A—S1—C2—C3	−0.32 (18)	C11—C12—C13—C14	13.8 (3)
S1—C2—C3—C3A	0.4 (2)	C3—C12—C13—C14	−165.47 (19)
S1—C2—C3—C12	−175.16 (15)	C12—C13—C14—C15	−19.8 (2)
C4—C3A—C3—C2	−179.9 (2)	C12—C11—C15—C14	−10.9 (3)
C7A—C3A—C3—C2	−0.4 (2)	C1—C11—C15—C14	173.7 (2)
C4—C3A—C3—C12	−4.4 (3)	C13—C14—C15—C11	18.7 (2)
C7A—C3A—C3—C12	175.13 (19)	O21—C21—C22—C23	−179.9 (3)
C7A—C3A—C4—C5	−0.9 (3)	O29—C21—C22—C23	0.3 (5)
C3—C3A—C4—C5	178.6 (2)	C21—C22—C23—C24	0.9 (5)
C3A—C4—C5—C6	0.1 (4)	C31—N5—C24—O28	179.9 (2)
C4—C5—C6—C7	0.5 (4)	C1—N5—C24—O28	−2.5 (3)
C4—C3A—C7A—C7	1.3 (3)	C31—N5—C24—C23	0.6 (3)
C3—C3A—C7A—C7	−178.3 (2)	C1—N5—C24—C23	178.22 (19)
C4—C3A—C7A—S1	179.73 (15)	C22—C23—C24—O28	3.1 (4)
C3—C3A—C7A—S1	0.1 (2)	C22—C23—C24—N5	−177.6 (2)
C2—S1—C7A—C7	178.4 (2)	C24—N5—C31—C36	102.1 (2)
C2—S1—C7A—C3A	0.09 (16)	C1—N5—C31—C36	−75.6 (2)
C5—C6—C7—C7A	−0.1 (4)	C24—N5—C31—C32	−79.3 (3)
C3A—C7A—C7—C6	−0.8 (3)	C1—N5—C31—C32	103.1 (2)
S1—C7A—C7—C6	−178.9 (2)	C36—C31—C32—C33	−1.8 (3)
N5—C1—C11—C12	117.2 (2)	N5—C31—C32—C33	179.64 (19)
N5—C1—C11—C15	−68.1 (3)	C31—C32—C33—C34	1.8 (3)
C1—C11—C12—C3	−7.6 (4)	C32—C33—C34—C35	−0.4 (4)
C15—C11—C12—C3	177.3 (2)	C33—C34—C35—C36	−1.0 (4)
C1—C11—C12—C13	173.2 (2)	C32—C31—C36—C35	0.4 (3)
C15—C11—C12—C13	−1.9 (3)	N5—C31—C36—C35	179.0 (2)
C2—C3—C12—C11	−64.0 (3)	C34—C35—C36—C31	1.0 (4)
C3A—C3—C12—C11	121.0 (2)		

### Hydrogen-bond geometry (Å, º)

*Cg*3 and *Cg*4 are the centroids of the benzene ring (C3A/C4–C7/C7A) of the nine-membered ring system (S1/C2-C3/C3A/C4–C7/C7A) and the phenyl ring (C31–C36), respectively.

**Table:**

*D*—H···*A*	*D*—H	H···*A*	*D*···*A*	*D*—H···*A*
O29—H29···O28	0.93 (4)	1.60 (4)	2.510 (3)	164 (4)
C32—H32*A*···O21^i^	0.93	2.63	3.556 (3)	171
C2—H2*A*···*Cg*4	0.93	2.72	3.579 (2)	154
C33—H33*A*···*Cg*3^ii^	0.93	2.54	3.408 (3)	155
C33—H33*A*···*Cg*5^ii^	0.93	2.64	3.353 (3)	133
C36—H36*A*···*Cg*3^iii^	0.93	2.79	3.535 (3)	138
C36—H36*A*···*Cg*5^iii^	0.93	2.75	3.623 (3)	157

Symmetry codes: (i) −*x*+1, −*y*, −*z*; (ii) −*x*+2, −*y*+1, −*z*+1; (iii) −*x*+1, −*y*+1, −*z*+1.
